# Transcriptomic Analysis of Diabetic Erectile Dysfunction Rats After Red Blood Cell Exosome Treatment

**DOI:** 10.3390/genes16070768

**Published:** 2025-06-29

**Authors:** Yantong Lv, Biaohu Quan, Xinyue Liu, Qichao Cui, Xi-Jun Yin

**Affiliations:** 1 Jilin Provincial Key Laboratory of Transgenic Animal and Embryo Engineering, Yanbian University, Yanji 133002, China0000008591@ybu.edu.cn (B.Q.);; 2 Laboratory Animal Center, Yanbian University, Gongyuan Street, Yanji 133002, China

**Keywords:** erectile dysfunction, diabetes, collagen deposition, transcriptomic

## Abstract

Background: As the prevalence of diabetes continues to rise each year, increasing attention is focused on its complications, including erectile dysfunction (ED). However, effective therapeutic agents for diabetes mellitus erectile dysfunction (DMED) are often inadequate. Exosomes, which are extracellular vesicles containing proteins and microRNAs, demonstrated remarkable capabilities in modulating pathophysiological processes related to tissue repair, anti-inflammatory responses, and immune regulation. Methods: Transcriptomic analysis was conducted to investigate gene alterations and associated pathways in the penile smooth muscle of DMED rats, both before and after exosome treatment. And the genes (*Rxra*, *PPAR-γ*, and *CPt1a*) related to the PPAR pathway were verified through qRT-PCR. Results: Results show that 13,947 genes were expressed in both the DMED group and the Exo group. Analysis of gene ontology (GO) and Kyoto Encyclopedia of Genes and Genomes (KEGG) pathways revealed significant enrichment in the Exo group for molecular pathways including PPAR and cAMP signaling. These genes are primarily involved in immune regulation and collagen deposition biological processes within the smooth muscle of the penis in DMED rats. Conclusions: Transcriptome analysis revealed important genes and pathways that regulate various biological processes. These findings offer a novel approach for decreasing collagen deposition in this tissue.

## 1. Introduction

Diabetes mellitus is a metabolic disorder characterized by elevated blood sugar levels. Epidemiological surveys indicate that by 2030, approximately 25 percent of the global population will be affected by this chronic condition [1]. As the morbidity and mortality associated with DM rise, the complication rate also increases, posing a significant threat to human health. One of the complications of diabetes is erectile dysfunction, defined as the persistent inability to achieve and maintain an erection sufficient for satisfactory sexual intercourse [2]. Diabetes can lead to erectile dysfunction by disrupting the structural integrity and function of the corpus cavernosum, promoting apoptosis of smooth muscle cells and increasing collagen deposition. Currently, the standard treatment of DMED is oral PDE5i [3,4], which inhibits the degradation of cyclic guanosine monophosphate in the smooth muscle cells of the penis, thereby promoting sustained relaxation of the smooth muscle and improving erectile function in ED patients. However, the therapeutic effectiveness of PDE5i in patients with DMED is reduced due to diabetes-induced endothelial dysfunction and impaired nitric oxide bioavailability, which fundamentally compromise the vasodilatory efficacy of PDE5i in penile resistance arteries [5]. Therefore, developing effective and non-toxic DMED treatments remains an urgent priority.

Exosomes are a subtype of extracellular vesicles, with diameters ranging from 30 to 100 nm [6]. They are known to be secreted by various bodily fluids, including blood, semen, urine, milk, and tears [7]. Functioning as sophisticated biological couriers, exosomes facilitate the horizontal transfer of bioactive molecules, such as microRNAs (miRNAs), messenger RNAs (mRNAs), and proteins, across cellular microenvironments. Growing evidence supports their therapeutic potential for treating erectile dysfunction, being attributed to their favorable biocompatibility and transport capabilities [8,9,10].

In this study, transcriptome sequencing technology was employed to investigate the gene expression patterns and co-expression trends of differentially expressed genes (DEGs) in the DMED group and the Exo group. GO and KEGG enrichment analyses of DEGs identified key genes and biological pathways modulated by exosomes within the penile smooth muscle of DMED rats. These findings will provide a foundation for further understanding the mechanisms by which exosomes affect the smooth muscle of the penis in DMED rats.

## 2. Materials and Methods

### 2.1. Experimental Animals

Fifteen six-week-old male Sprague–Dawley rats, aged 6–8 weeks and weighing 200–230 g, were purchased from the Laboratory Animal Center of Yanbian University in Yanji, Jilin Province. They were exposed to a consistent, daily 12 h light and dark cycle and quantitative food and water, a room temperature of 22–25 °C, and relative humidity of 45–55%. All experiments comply with the Code of Ethics for Animal Experiments (YD20240809001).

### 2.2. Main Instruments and Reagents

Three-dimensional culture vial (Corning, New York, NY, USA), STZ (MedChemexpress, Monmouth Junction, NJ, USA), apomorphine (MedChemexpress, NJ, USA), CD63 antibody (Abcam, Cambridge, UK), CD81 antibody (Abcam, Cambridge, UK), Col-1 antibody(Abcam, Cambridge, UK), Masson staining kit (Solarbio, Beijing, China), PKH67 fluorescent dye kit (Solarbio, Beijing, China), and Haematoxylin and eosin (H&E) staining kit(Solarbio, Beijing, China).

### 2.3. Isolation, Identification and Uptake of Red Blood Cell Exosomes

Whole blood samples from healthy pigs were collected and centrifuged to isolate plasma. The samples were then centrifuged at 3000× *g* for 4 min to eliminate impurities. The red blood cells were transferred to a three-dimensional rotating flask and cultured in 500 mL of NCSU medium for 48 h. Following the culture period, the collected culture medium was subjected to a series of centrifugation steps: first at 300× *g* for 10 min, then at 2000× *g* for 10 min, followed by 10,000× *g* for 30 min, and finally at 100,000× *g* for 90 min to isolate red blood cell exosomes, which were subsequently stored at −80 °C. Exosome size was determined by nanoparticle tracking analysis. Morphology was examined using transmission electron microscopy, while Western blot confirmed expression of the exosomal markers CD81 and CD9.

For tracking studies, exosomes were labeled with PKH67 fluorescent dye at room temperature in the dark for 1 h, and the excess dye was removed through ultracentrifugation at 100,000× *g*. Labeled exosomes were injected into the corpora cavernosa of SD rats. Animals were euthanized at 24 h and 48 h post-injection. Penile tissue was then extracted and subjected to frozen sectioning to observe the uptake of exosomes. Sections were counterstained with DAPI.

### 2.4. Establishment and Identification of DMED Rats Model

After a two-week acclimation period, 15 Sprague–Dawley (SD) rats were intraperitoneally injected with streptozotocin (60 mg/kg). Blood glucose levels (BSL) were measured using a glucose meter on day 60 following the STZ injection [11,12]. A BSL exceeding 16.7 mmol/L was considered indicative of diabetes in rats, and a total of 10 DM rats were successfully established. In total, 10 DM rats were weighed and placed in a quiet, dimly lit observation box for 10 min. The prepared APO was then injected subcutaneously into the loose skin of the rats’ necks at a dosage of 100 μg/kg, and penile erection was observed for 30 min. Erection was defined as the enlargement of the penis, with the glans becoming exposed. A total of 8 rats that did not exhibit erections in the DM rat model were classified as DMED model rats.

### 2.5. Grouping and Administration of Animals

Eight male Sprague–Dawley rats were divided into two groups: the DMED group (*n* = 4) and the RBC-exo group (*n* = 4). Each rat in the DMED group was injected with 200 μL of phosphate-buffered saline (PBS), while the rats in the RBC-exo group received 20 μL of 10^10^ particles of RBC-exo dissolved in PBS, for a total volume of 200 μL. All treatments were administered via injection into the penis every three days for a duration of three weeks. The control group consisted of DMED rats without modeling (*n* = 4).

### 2.6. Tissue Sampling and Testing

After anesthesia, the penises removed from all SD rats were divided into three sections: the first section was stored at −80 °C for subsequent experiments, the second section was immediately pre-frozen using liquid nitrogen and isopentane before being sliced, and the final section was utilized for sequencing analysis.

#### 2.6.1. Observation of Masson Staining and H&E Staining

The frozen samples were sectioned into 12 μm-thick slices using a microtome and stained according to the protocol provided by the Masson staining kit and H&E staining. Images were captured with a fluorescence microscope, and the surface area ratio of smooth muscle (red) to collagen (blue) in the cavernous body of the penis was calculated using ImageJ software V1.8.0.

#### 2.6.2. Immunofluorescence Staining

The frozen sections of penile tissue were washed with PBS and dried. They were then incubated with 5% goat serum at room temperature for 1 h, followed by incubation with the primary antibody at 4 °C overnight. The nuclei were stained with DAPI solution and incubated at room temperature for 15 min, protected from light. After cleaning with PBS, moisture was controlled, and an anti-fade mounting medium was applied to seal the coverslip, ensuring that the slide remained wet. A confocal laser microscope was used to observe and photograph the samples. ImageJ software was employed to randomly select different azimuthal regions for statistical analysis.

#### 2.6.3. Western Blot

The rat penis samples were weighed, and the protein was extracted using RIPA lysis buffer. The concentration of the protein was determined and quantified using a BCA assay. The protein samples were then denatured by boiling. A 12% separation gel and a 5% stacking gel were prepared based on the amount of protein samples. The electrophoresis apparatus was assembled, and the conditions were set to 80–90 V for 20 min, followed by electrophoresis at 100–110 V for 1 h. ImageJ software was utilized to analyze the bands, and the results were processed digitally. All results were normalized against the internal reference protein β-actin.

#### 2.6.4. Quantitative Real-Time Polymerase Chain Reaction (qRT-PCR)

Total RNA was isolated from rat penis (ED group and Exo group) using Trizol reagent(TIANGEN, Beijing, China). Complementary DNA was prepared by reverse transcription PCR using the StarScript III RT Kit (GenStar, Beijing, China). The reaction conditions were 37 °C 2 min, 85 °C 2 min, and 4 °C ∞. Primer sequences were listed in Table 1. Gene expression values were normalized to GAPDH and represented as fold change of the ED group.

### 2.7. Transcriptomic Analysis

RNA-seq analysis was conducted on the penises of eight DMED SD rats. In brief, the total quantity and integrity of RNA were assessed from the penile samples of the DMED SD rats using the Agilent 2100 Bioanalyzer (Agilent, Santa Clara, CA, USA). The image data of the sequencing fragments, captured by the high-throughput sequencer, were converted into sequence data using CASAVA base recognition. Using DESeq2, genes with adjusted *p*-values ≤ 0.05 were identified as differentially expressed genes (DEGs). The criteria for identifying DEGs were set as log2|(FC) |> 1 and adjusted *p*-value < 0.05. The gene expression level was quantified by fragments per kilobase of transcript per million mapped reads (FPKM) which could normalize the number of reads and the length of transcripts. Genes differential expression analysis was performed by DESeq2 software V3.21 between two different groups (and by edgeR between two samples). The genes with the parameter of q-value < 0.05 and a fold change (FC) ≥ 2 or FC ≤ 0.5 were considered differentially expressed genes. The differentially expressed genes were then subjected to enrichment analysis of GO functions and KEGG pathways. GO annotation and KEGG analyses were performed using the NovoMagic platform. A Sankey bubble diagram and correlation assessment diagram for the different groups were generated using the online platform https://www.bioinformatics.com.cn (accessed on 10 December 2024), an online platform for data analysis and visualization. According to the KEGG PATHWAY Database (https://www.kegg.jp/kegg/pathway.html, accessed on 10 December 2024), obtain the secondary classification results of KEGG. 

### 2.8. Data Analysis

All experiments were conducted a minimum of three times. Data are presented as the mean ± standard deviation, analyzed using Student’s *t*-test, with *p* < 0.05 considered statistically significant (*n* = 3).

## 3. Result

### 3.1. Exosome Isolation and Identification

**Figure 1:**
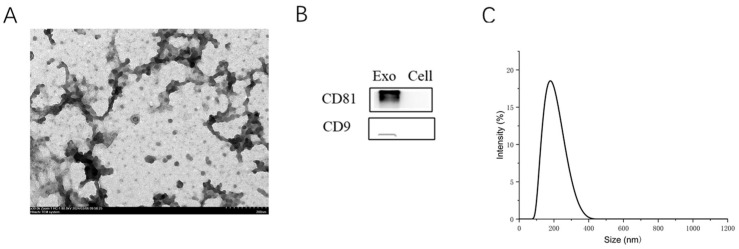
Characterization of exosome. (**A**) Representative transmission electron micrograph of RBC-exo; (**B**) Western blot results show positive expressions of CD81 and CD63 proteins in RBC-exo. (**C**) Particle size distribution of RBC-exo measured by NanoSight system.

Transmission electron microscopy revealed that RBC-exo exhibited a cupped morphology (Figure 1A). Western blot analysis confirmed the presence of positive exosomal surface markers CD9 and CD81 in RBC-exo (Figure 1B). Utilizing the NanoSight system, the average diameter of RBC-exo was determined to be 174 nm (Figure 1C). Additionally, exosome fluorescence in penile tissue was observed to peak at 24 h post-ingestion and subsequently declined by 48 h (Supplementary Figure S1).

### 3.2. Collagen Deposition in the Penile Cavernous Bodies of DMED Rats Decreased Following Exosome Treatment

**Figure 2:**
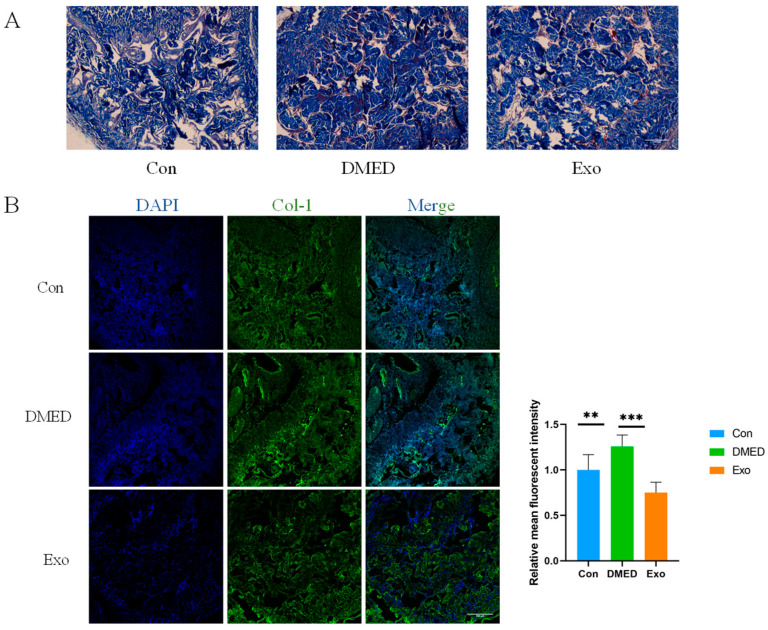
DMED rat penile cavernous collagen deposition content decreased after exosome treatment. (**A**) Masson staining. Smooth muscle (red), collagen (blue). Scale = 500 μm. (**B**) Immunofluorescence staining in control, DMED, and exosome groups. Scale = 200 μm. ** *p* < 0.01, and *** *p* < 0.001.

The results of Masson staining and immunofluorescence staining indicate that, compared to the control group, the collagen content in the penile cavernous body of rats in the DMED group increased, while the smooth muscle area showed no significant change (Figure 2A). Additionally, collagen deposition in the DMED group was significantly elevated. After 21 days of exosome treatment, immunofluorescence staining revealed that, compared to the DMED group, collagen deposition in the penile cavernous body of rats in the exosome group was significantly reduced (Figure 2B).

### 3.3. Expression Profiles of Differentially Expressed Genes (DEGs) in the DMED Group and Exosome Group

**Figure 3:**
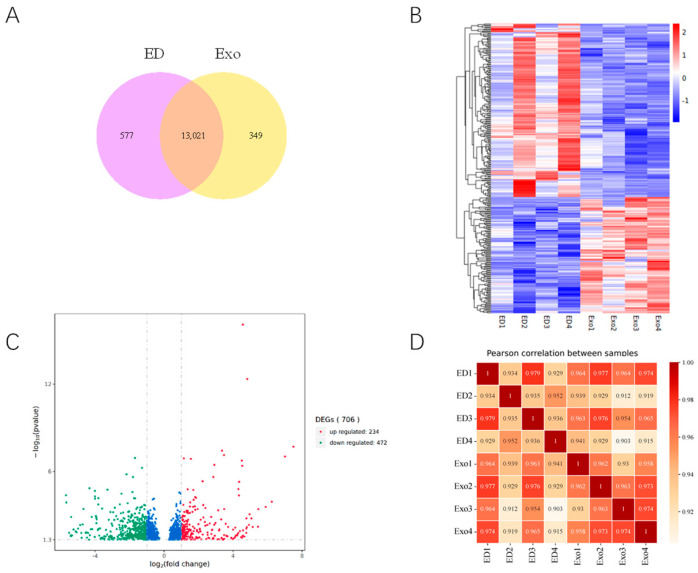
DEGs expression profiles during ED and Exo groups. (**A**) Venn diagram of DEGs in ED and Exo groups. (**B**) Heatmap of DEGs. Red indicates that the gene is expressed at high levels, and blue indicates lower expression. (**C**) Volcano plots of the DEGs during ED and Exo groups. The blue dots in the middle represent genes with no significant differences. (**D**) Correlation assessment of the ED and Exo groups (*n* = 4). The Pearson correlation coefficient R (Pearson’s correlation coefficient) was used to evaluate reproducibility. A value of 1 (R^2^) indicates better reproducibility between the two samples. Red indicates high correlation, and yellow indicates lower correlation.

Following the experiments described above, transcriptome sequencing was conducted to further investigate the gene expression profiles in the DMED and Exo groups. The results indicate that a total of 13,947 genes were expressed in both the DMED and Exo groups. Additionally, 577 genes were exclusively expressed in the DMED group, while 349 genes were uniquely expressed in the Exo group (Figure 3A). A total of 706 DEGs were identified between the Exo and DMED groups, of which 234 were upregulated and 472 were downregulated (Figure 3B,C). Pearson correlation analysis revealed a high correlation index between the DMED and Exo groups (Figure 3D).

### 3.4. The Biological Activities Decreased in the ED Group Rats

**Figure 4:**
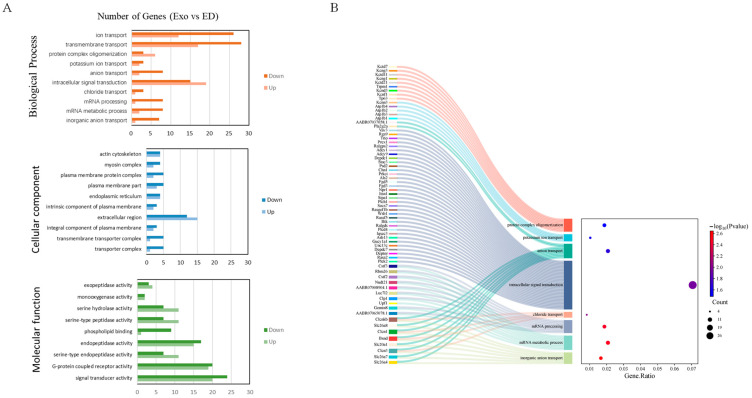
GO analysis of genes that were differentially expressed during ED and Exo groups. (**A**) GO analysis of genes in three categories (biological process, cellular component, and molecular function). Horizontal axis: number of differentially expressed genes annotated to the terms. Vertical axis: GO terms and classifications. (**B**) Dot plot of the Go pathway enrichment analysis. The horizontal axis represents the gene ratio, while the vertical axis represents the enriched pathway name. The color scale indicates different thresholds of the *p*-value, and the size of the dot indicates the number of genes corresponding to each pathway.

**Figure 5:**
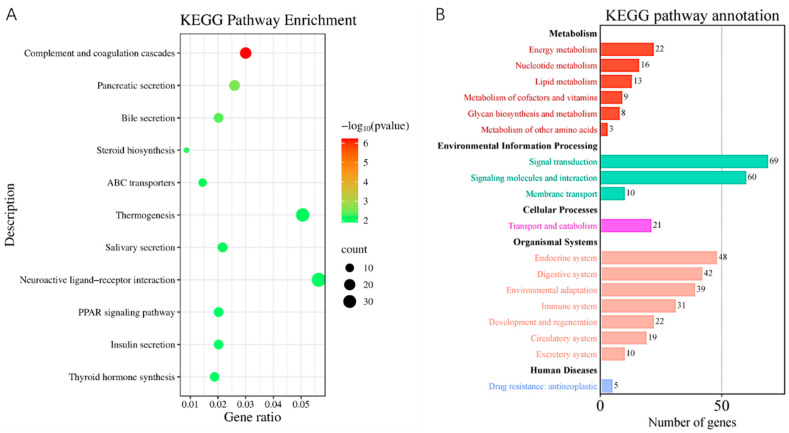
KEGG pathway enrichment of genes. (**A**) KEGG pathway enrichment of genes that were differentially expressed during ED and Exo groups. (**B**) Secondary classification of KEGG pathway enrichment results. Dot plot of the KEGG pathway enrichment analysis. The horizontal axis represents the gene ratio, while the vertical axis represents the enriched pathway name. The color scale indicates different thresholds of the *p*-value, and the size of the dot indicates the number of genes corresponding to each pathway.

The GO functional classification of the DEGs revealed that the downregulated genes were more significantly enriched in most GO terms related to the ED group compared to the upregulated genes. Biological processes (BP) include ion transport, transmembrane transport, mRNA processing, mRNA metabolic processes, and inorganic anion transport. Cellular components (CC) consist of integral components of the plasma membrane, transporter complexes, transmembrane transporter complexes, and parts of the plasma membrane. Molecular functions (MF) encompass signal transducer activity, phospholipid binding, and calcium-dependent phospholipid binding (Figure 4A). As shown in Figure 4B, the left side of the figure presents a Sankey diagram, which illustrates the genes associated with each pathway, while the right side displays a conventional bubble diagram. The KEGG analysis was conducted to identify the enriched pathways of the target genes. The enriched categories of KEGG pathways are depicted in Figure 5A. Results indicate that the complement and coagulation cascades pathway, ABC transporters, steroid biosynthesis, neuroactive ligand–receptor interaction, PPAR signaling pathway, and cAMP signaling pathway were significantly enriched in the Exo group, providing molecular mechanisms for DMED treatment. According to the KEGG secondary classification enrichment results, the majority of genes were enriched in signal transduction pathways, signaling molecules, interaction pathways, and the endocrine system. Furthermore, five genes in the Exo group were associated with drug resistance, specifically within the antineoplastic pathway (Figure 5B).

**Figure 6:**
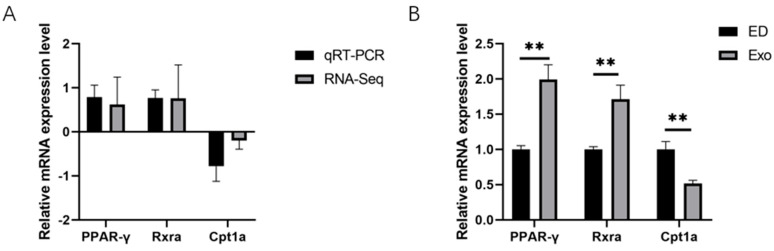
The expression levels of genes related to the PPAR pathway. (**A**,**B**) The expression levels of some important Rxra, PPAR-γ, and CPt1a were verified by qRT‒PCR. Significant differences are represented by ** *p* < 0.01.

To verify the mRNA expression levels of the DEGs that were identified via RNA-Seq, three important DEGs were randomly selected, and their expression levels were verified via qRT‒PCR. As shown in Figure 6A,B, the expression trends of these three genes in the ED group and Exo group, as determined by qRT‒PCR, were consistent with the RNA‒Seq results. The qRT‒PCR results indicate that the mRNA expression levels of CPt1a were significantly decreased (0.51-fold in the exosome group, whereas the levels of Rxra and PPAR-γ were significantly increased (1.71-fold and 1.99-fold of those in the exosome group, respectively). In addition, oxidative stress markers—inducible nitric oxide synthase, 1.9-fold (iNOS) and endothelial nitric oxide synthase, 2.38-fold (eNOS)—were significantly lower in the exosome group compared to the ED group.

## 4. Discussion

Erectile dysfunction is a significant health concern for men with diabetes, and ED resulting from diabetes mellitus is particularly challenging to manage. Recent studies confirmed that exosomes are bioactive substances that can treat ED through paracrine therapy. These exosomes are involved in various physiological and pathological processes, including the reduction in collagen deposition [13,14,15].

Penile erectile dysfunction refers to a male reproductive disorder characterized by the inability to achieve or maintain an adequate erection. The pathogenesis of DMED is complex, involving factors such as neuropathy, endothelial cell dysfunction, structural and functional changes in the spongy smooth muscle, hormonal alterations, and other contributing aspects [16,17,18]. One of the critical factors is the disruption in the structure and function of the cavernous body, characterized by impaired smooth muscle relaxation and excessive collagen deposition in the smooth muscle of the penis due to hyperglycemia. In this experiment, Masson staining and immunofluorescence staining revealed that, compared to the control group, rats in the DMED group exhibited increased collagen deposition in penile smooth muscle. This experiment has the limitation of a small sample size. However, based on previous papers and the statistical analysis of this study, there are differences, and we believe the experiment is reasonable.

Exosomes play an active role in tissue repair, anti-inflammation, immune regulation, and other biological processes, making them valuable in disease intervention and therapeutic applications. Increasing evidence suggests that exosomes are essential for restoring erectile function in patients with diabetic erectile dysfunction (DMED). Exosomes derived from cavernous smooth muscle cells have been shown to alleviate erectile dysfunction by inhibiting cavernous fibrosis and modulating the nitric oxide/cyclic guanosine monophosphate (NO/cGMP) pathway [15]. In this experiment, RBC-exo was administered to DMED rats. The results indicate that the injection of red blood cell exosomes into the corpus cavernosa of the penis significantly reduced collagen deposition in the smooth muscle of DMED rats. This finding aligns with previous research demonstrating improvements in cavernous fibrosis and a reduction in collagen deposition in DMED rats following treatment with human urine-derived stem cell exosomes [19]. In addition, we observed that the fluorescence intensity of PKH-67-labeled exosomes gradually decreased 48 h after uptake by penile tissue. Consequently, we administered exosome injections every three days to maintain therapeutic efficacy.

GO analysis revealed significant enrichment in transmembrane transport, ion transport, signal transducer activity, and transporter complex pathways. The DEGs indicated that downregulated genes were more prevalent than upregulated genes, suggesting that they may not be the primary factors through which exosomes influence collagen deposition. Additionally, the KEGG analysis identified enrichment in the complement and coagulation cascades, as well as the PPAR signaling pathway, within the Exo group. Peroxisome proliferator-activated receptors (PPARs) are part of the same category as thyroid hormones, vitamin A-like receptors, steroid hormones, and vitamin D receptors. As members of the nuclear hormone receptor family, PPARs are widely distributed across various systems and organs in the human body. PPAR agonists activate PPAR-γ-mediated pro-fibrotic signals, which help reduce excessive collagen deposition in the skin [20,21,22]. Therefore, we speculate that the marked reduction in collagen deposition in the cavernous smooth muscle of DMED rats may be associated with the activation of the PPAR signaling pathway. Therefore, we verified the important genes related to the PPAR pathway (CPt1a, Rxra, and PPAR-γ), and obtained results consistent with those from RNA-seq.

To further explore whether exosomes affect oxidative stress, oxidative stress markers (iNOS and eNOS) were detected. During the process of erection, the release of neurotransmitters—particularly nitric oxide (NO), which is synthesized by nitric oxide synthase (NOS)—plays a crucial role in relaxing the smooth muscle [23]. However, under pathological conditions, when NOS loses its coupling, it can lead to erectile dysfunction (ED) and local oxidative stress. In the uncoupled state, the various subtypes of NOS (eNOS, nNOS, and iNOS) produce superoxide radicals instead of nitric oxide, further exacerbating vascular and endothelial dysfunction [24]. The results show that the iNOS and eNOS in the exosome group were significantly reduced, indicating that exosomes had a certain antioxidant stress effect.

In conclusion, transcriptome analysis revealed important genes and pathways that regulate various biological processes. Key molecular pathways, such as the complement and coagulation cascades and the PPAR signaling pathway, were enriched in the Exo group. These findings enhance our understanding of the processes involved in DMED and the underlying mechanisms, providing a theoretical basis for the effective treatment of DMED.

## Figures and Tables

**Table 1:** PCR primer sequence.

Gene	Primer Sequence (5′-3′)	Length (bp)
Rxra	Forward:ATTTCCTGCCGCTCGACTT Reverse:GCTGATGACCGAGAAGGGTG	209
Cpt1a	Forward:GGCTTGGGACTTGGGCTTAC Reverse:CTTCTCTGCAACCCGGTAGG	158
PPAR-γ	Forward:GCAGAAACTGGGAGTAGCCTG Reverse:ATGGCATCTCTGTGTCAACCA	223
GADPH	Forward:CGTAAAGACCTCTATGCCAACA Reverse:CGGACTCATCGTACTCCTGCT	76

## Data Availability

The original contributions presented in the study are included in the article/Supplementary Materials. Further inquiries can be directed to the corresponding author.

## Supplementary Materials

The following supporting information can be downloaded at: https://www.mdpi.com/article/10.3390/genes16070768/s1, Figure S1: PKH67-labeled exosomes by SD rats at 24 h and 48 h. Figure S2: DMED rat penile cavernous collagen deposition content by HE staining. Figure S3: Immunofluorescence staining for SMA to identified as CCSMCs. Figure S4:qRT-PCR detect oxidative stress-related indicators.
