# Supplementary material for: Transcriptomic Analysis of Diabetic Erectile Dysfunction Rats After Red Blood Cell Exosome Treatment
Source: Genes (Basel). 2025 Jun 29;16(7):768. doi: 10.3390/genes16070768 (PMC12295123; doi:10.3390/genes16070768)
Supplement: Supplementary file 1 [file genes-16-00768-s001.zip › genes-3699680-supplementary.pdf]

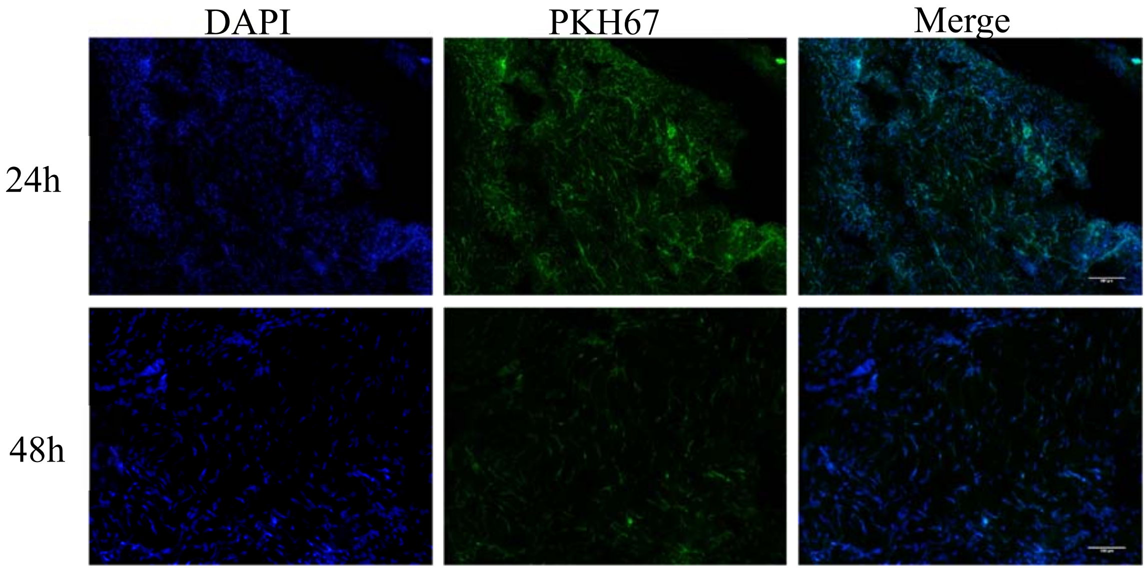

Supplementary Figure S1. Images showing the uptake of PKH67-labeled exosomes by SD rats at 24 hours and 48 hours.

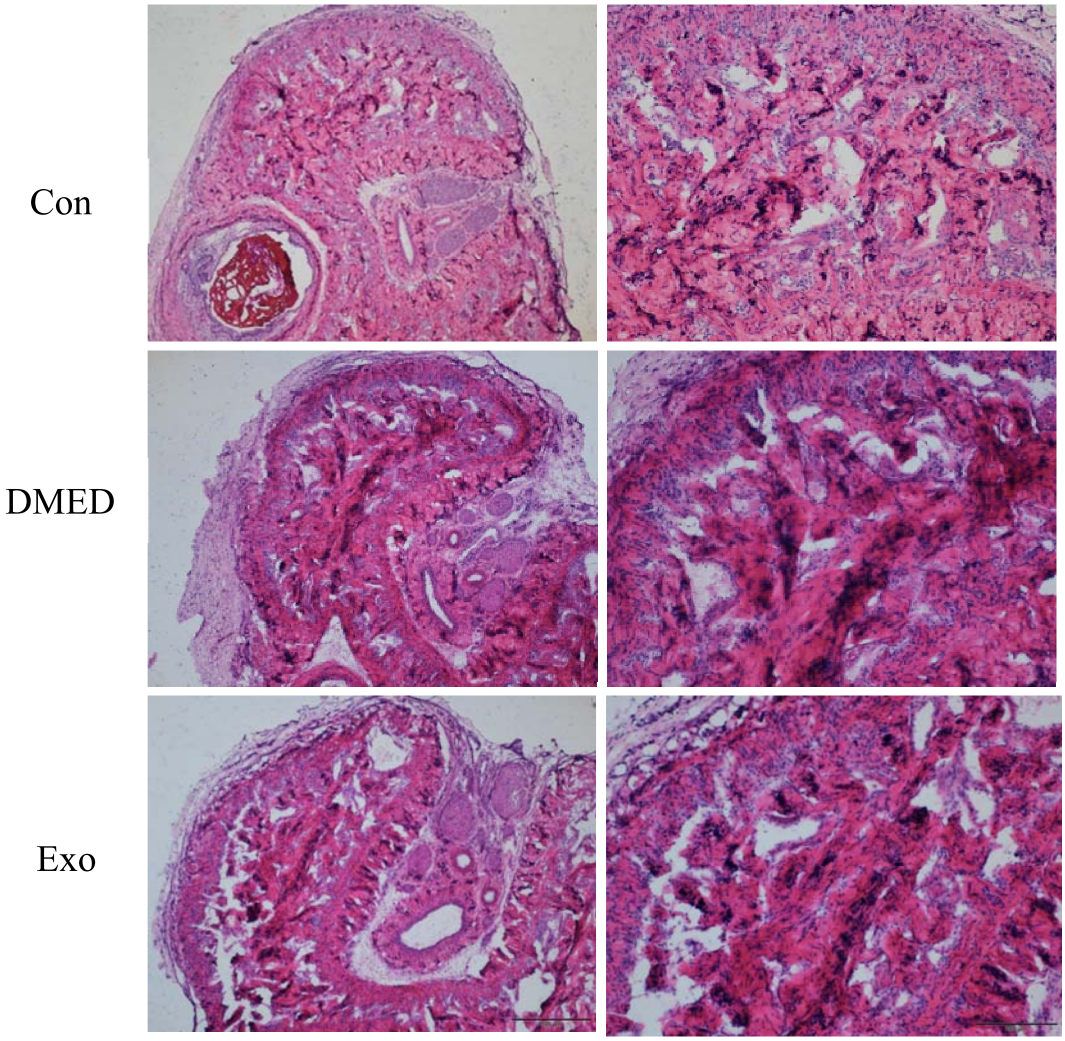

Supplementary Figure S2. DMED rat penile cavernous collagen deposition content by HE staining.

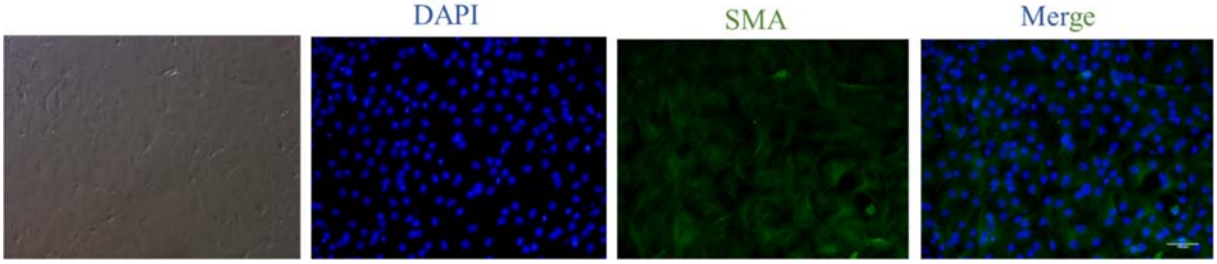

Supplementary Figure S3. Immunofluorescence staining for SMA to identified as CCSMCs. Scale bar =100µm.

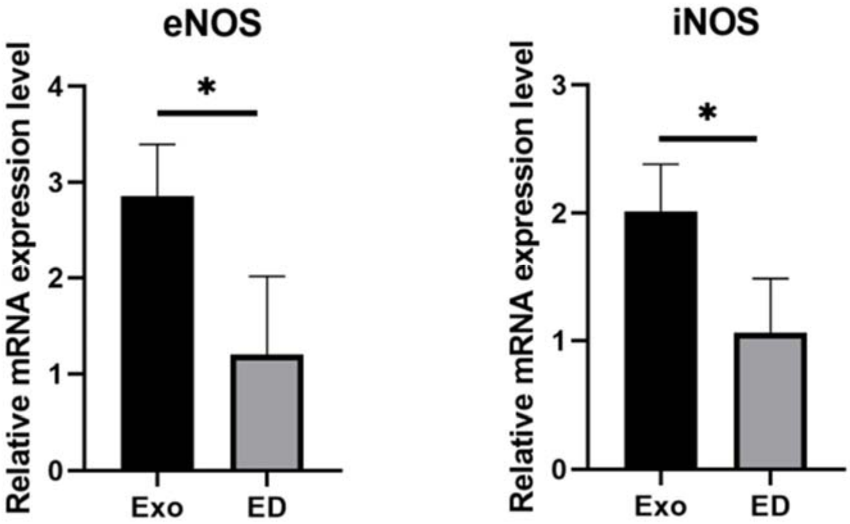

Supplementary Figure S4. qpcr detect oxidative stress-related indicators. (A) eNOS (endothelial nitric oxide synthase) gene (B) iNOS (Inducible nitric oxide synthase) gene.

Supplementary Material

A 25T sterile culture flask containing rat penile tissue blocks was prepared for adherent culture. Initially, 2 ml of DMEM supplemented with 15% serum was added. After the tissue adhered to the flask wall, an additional 1 ml of culture medium was supplemented. Once a significant number of cells had migrated out of the tissue mass, the tissue was removed. The culture was rinsed with DPBS, and then 3 ml of complete culture medium was added to continue the culture process. The differential adherent method was employed for cell purification. Finally, the isolated cells were identified using immunofluorescence staining for smooth muscle actin (SMA).
